# Accelerated synthesis of energetic precursor cage compounds using confined volume systems

**DOI:** 10.1038/s41598-021-02945-1

**Published:** 2021-12-16

**Authors:** Hilary M. Brown, Karan R. Doppalapudi, Patrick W. Fedick

**Affiliations:** Chemistry Division, Naval Air Warfare Center Weapons Division (NAWCWD), United States Navy Naval Air Systems Command (NAVAIR), China Lake, CA 93555 USA

**Keywords:** Chemistry, Analytical chemistry, Green chemistry, Chemical synthesis

## Abstract

Confined volume systems, such as microdroplets, Leidenfrost droplets, or thin films, can accelerate chemical reactions. Acceleration occurs due to the evaporation of solvent, the increase in reactant concentration, and the higher surface-to-volume ratios amongst other phenomena. Performing reactions in confined volume systems derived from mass spectrometry ionization sources or Leidenfrost droplets allows for reaction conditions to be changed quickly for rapid screening in a time efficient and cost-saving manner. Compared to solution phase reactions, confined volume systems also reduce waste by screening reaction conditions in smaller volumes prior to scaling. Herein, the condensation of glyoxal with benzylamine (BA) to form hexabenzylhexaazaisowurtzitane (HBIW), an intermediate to the highly desired energetic compound 2,4,6,8,10,12-hexanitro-2,4,6,8,10,12-hexaazaisowurtzitane (CL-20), was explored. Five confined volume systems were compared to evaluate which technique was ideal for forming this complex cage structure. Substituted amines were also explored as BA replacements to screen alternative cage structure intermediates and evaluate how these accelerated techniques could apply to novel reactions, discover alternative reagents to form the cage compound, and improve synthetic routes for the preparation of CL-20. Ultimately, reaction acceleration is ideal for predicting the success of novel reactions prior to scaling up and determining if the expected products form, all while saving time and reducing costs. Acceleration factors and conversion ratios for each reaction were assessed by comparing the amount of product formed to the traditional bulk solution phase synthesis.

## Introduction

Organic reactions are significantly accelerated in confined volume systems, such as microdroplets^1–10^, Leidenfrost droplets^11–14^, and thin films^15–19^, when compared to their bulk solution phase reaction counterparts. Acceleration was first observed in electrospray microdroplets when Augusti et al. performed Eberlin transacetalization reactions under ambient conditions^20,21^. Reaction rates are increased in confined volume systems due to partially solvated reactants, with decreased energy barriers caused by solvent evaporation affecting reactant concentration, extreme pH in droplet environment, fast diffusion and mixing within the droplet, and larger surface-to-volume ratios^15,16,22–29^. Due to these unique conditions, with certain reactions, catalysts can be eliminated from the reaction while still producing product^30,31^. Compared to bulk reactions, the formation of reaction products can be accelerated in microdroplets, producing product roughly 10 to 10^6^ times faster^24,32^. Therefore, reaction acceleration can be used to determine if a reaction will form the expected products and predict the success of the reaction before scaling up, saving time and money^33,34^.

The speed of these reactions enables one to follow reaction kinetics, screen catalysts and reaction conditions, investigate intermediates, and explore degradation processes^1,12,32,35^. In addition to rapid screening, Cooks et al*.* has shown that product can be produced at a rate of 100 mg per hour using thin films, with the potential for larger product formation if the setup was multiplexed^2,36^. Cooks et al*.* also showed that reaction solvent can be recycled in a spray-based setup to allow for longer reactions time to increase product yield up to 3 g per hour^37^.

Modified mass spectrometry ionization sources, including electrospray (ESI)^6,15,33,35,38,39^, electrosonic spray ionization (ESSI)^2,3^, nano-electrospray ionization (nESI)^1,3^, easy ambient sonic-spray ionization (EASI)^40^, as well as desorption electrospray ionization DESI^13,41^, have been used to generate microdroplets for acceleration. Paper spray ionization (PSI) sources have also been used for reaction acceleration through thin films^17,32,42^. EASI utilizes gas to desolvate droplets as they travel from the source to the glass wool for collection. ESSI also uses desolvation gas but incorporates high voltage to generate charged droplets. nESI uses high voltage but no desolvation gas and through the use of a smaller diameter spray capillary, smaller droplets are also formed. Acceleration in PSI is attributed to thin films which are created on surfaces after reagents are deposited and the solvent evaporates^16^. Acceleration in thin films occurs due to the increased reagent concentration after the solvent has evaporated^18^. Leidenfrost droplets, while not formed through MS ionization sources, create confined volume droplets using extreme heat that is significantly higher than the boiling point of the solvent used^11,12^. These droplets have no net charge and levitate on an insulating vapor cushion. As the solvent evaporates, additional solvent is added dropwise to maintain the droplet size as the reaction progresses.

A wide variety of reactions have been explored using confined volume systems^28,43^, even the thermal decomposition of RDX, but these techniques have yet to be applied to the accelerated formation of energetic compounds^44^. 2,4,6,8,10,12-Hexanitro-2,4,6,8,10,12-hexaazaisowurtzitane, also known as CL-20, was first synthesized by Nielsen et al*.*^45,46^. It is one of the most powerful and stable energetic compounds^47^. Compared to other high energy explosives, including HMX, RDX, and PETN, CL-20 has superior performance with respect to detonation velocity, detonation pressure, and enthalpy of formation^48^. Due to the highly strained bond angles of the cage, strain energy is trapped within the molecule. CL-20 also contains six nitro groups giving the molecule an excellent oxidizer to fuel ratio. The energy released through oxidation combined with the inherent strain energy gives CL-20 its higher detonation velocity and enthalpy of formation^47^.

The structure of CL-20 is a polycyclic nitramine cage whose basic cage structure is formed from the condensation of glyoxal and benzylamine (BA) to produce hexabenzylhexaazaisowurtzitane (HBIW)^49,50^. The reaction for preparing HBIW is performed in an organic solvent, typically acetonitrile, and in the presence of an acid catalyst, typically formic acid (FA)^46,49,51^. The reaction mechanism for preparing HBIW involves several known intermediate structures that contain a dicarbinolamine or diimine functional groups^46,50,52^. The nitration step poses more difficulty due to the benzene rings; direct nitration by nitrolysis is not possible. Therefore, debenzylation is necessary before nitration^49^. Other methods that have been explored include using allylamine or sulfamates to replace BA in the condensation reaction and in turn eliminating the debenzylation step altogether, making the nitration step easier^48^.

Herein, we explore the preparation of HBIW in five confined volume systems to understand which technique is best suited for the formation of the complex cage structures. While EASI, ESSI, and nESI are all spray-based techniques, they vary in terms of desolvation gas, flow rate, and voltage application and were selected to explore the effects these factors have on acceleration. In microdroplets, the general trend is that reaction rate increases as droplet size decreases, therefore comparing acceleration rates from the larger droplets in ESSI or EASI with smaller droplets in nESI can provide insight into whether this is the case for the complex cage structure of HBIW^39,53^. Leidenfrost and thin films using PSI were also used to explore the effects of added heat and increased reaction surface area, respectively, on the acceleration rates of HBIW analogs.

**Figure 1:**
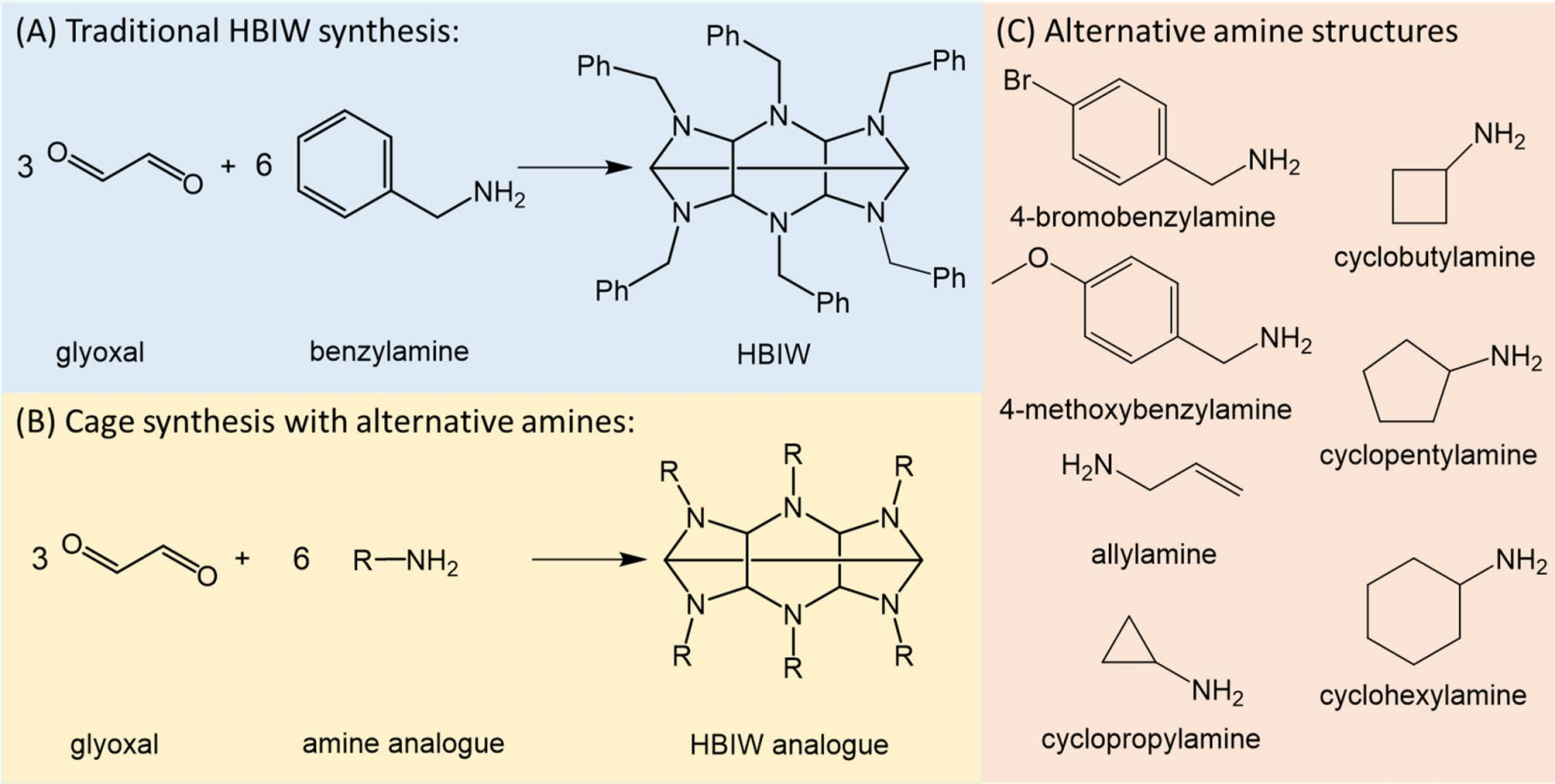
(**A**) Traditional synthesis of HBIW involves the condensation of glyoxal and BA to form HBIW which acts as the base cage structure. (**B**) Reaction scheme for the synthesis of alternative cage structures using amine analogues. (**C**) Structures of amine analogues used to replace BA as a reactant.

The screening capabilities of these techniques were also explored in order to evaluate if alternatives to HBIW can be produced, ultimately making the nitration simpler. Additionally, the unique microdroplet environment was examined to determine if the acid catalyst could be eliminated altogether. Eight different amines were screened including BA, allylamine (AA), bromobenzylamine (BrBA), methoxybenzylamine (mBA), cyclopropylamine (CPrA), cyclobutylamine (CBA), cyclopentylamine (CPA), and cyclohexylamine (CHA) (Fig. 1) as well as, various concentrations of FA ranging from 0 to 5%.

## Methods

### Reagents and materials

Glyoxal (40% wt in H_2_O), benzylamine (99%), cyclopropylamine, cyclobutylamine, cyclopentylamine, cyclohexylamine, and allylamine were purchased from Sigma Aldrich. 4-Bromobenzylamine was purchased from Oakwood Chemical. Formic acid (LiChropur) and 4-Methoxybenzylamine were purchased from EMD Millipore, and acetonitrile (HPLC LC–MS grade) was purchased from VWR chemical. Glyoxal stock solutions (10 mM) were made containing 0% formic acid (FA), 0.1% FA, 1% FA, 5% FA, and 10% FA. Solutions for each amine (8 total) were 20 mM. 500 µL of glyoxal with FA and 500 µL of amine variants were combined to create the reaction mixture, resulting in reaction solutions containing 10 mM amine, 5 mM glyoxal, and FA (0%, 0.05%, 0.5%, 2.5%, 5%).

### Reaction acceleration techniques and mass spectrometry analysis

Every reaction for each accelerated technique and corresponding bulks was conducted for 10 min. The individual setups and conditions for each of the accelerated techniques, along with pictorial representations can be found in the Supporting Information. The collected product and corresponding bulk solutions from each reaction were analyzed via nESI on a Thermo LTQ, except for PSI which was utilized as both the reaction surface and ionization source. Full scan mass spectra were collected in positive ionization mode. Capillary temperature was set to 400 °C and the spray voltage was 2.5 kV for nESI and 4 kV for PSI.

### Calculation of apparent acceleration factor and conversion ratio

Apparent acceleration factors (AAF) were calculated to compare the degree of acceleration across techniques and substituted amines used. The intensity of the amine starting material and formed product were obtained from the average spectra for both the accelerated system and the bulk. AAF were calculated using Equation S1 for each reaction. Each condition was analyzed in triplicate and the average AAF are shown in Table 1.

**Table 1:** Summary of the calculated AAFs and CRs for each accelerated technique, FA condition, and amine analogue.

Amine	% FA	EASI			+ 4 kV ESSI			− 4 kV ESSI			+ 3.5 kV nESI			− 3.5 kV nESI			LF			PSI		
		AAF	CR_droplet_	CR_bulk_	AAF	CR_droplet_	CR_bulk_	AAF	CR_droplet_	CR_bulk_	AAF	CR_droplet_	CR_bulk_	AAF	CR_droplet_	CR_bulk_	AAF	CR_droplet_	CR_bulk_	AAF	CR_droplet_	CR_bulk_
BA	0	11	4	0.5	13	6	0.6	20	9	0.7	2	2	1	6	0.8	0.2	8	6	1	464	26	1
	0.05	0.8	9	10	2	12	10	2	17	12	0.2	4	14	0.1	0.8	8	4	11	4	0.5	11	17
	0.5	4	5	1	3	6	2	5	6	2	0.2	0. 7	2	0.1	0.4	2	21	21	2	7	11	3
	2.5	65	3	0.1	92	2	0.02	71	4	0.08	8	0.2	0.03	6	0.3	0.1	9	0.7	0.1	135	10	0.2
	5	102	3	0.04	97	1	0.02	128	3	0.02	17	0.2	0.01	4	0.2	0.1	15	0.6	0.1	28	7	0.6
BrBA	0	4	0.8	0.2	6	0.4	0.1	3	0.3	0.1	4	0.2	0.1	4	0.3	0.1	9	9	2	10	2	1
	0.05	0.8	4	4	1	3	3	2	7	5	0.5	2	4	0.1	0.3	5	0.5	2	5	0.4	3	8
	0.5	2	3	1	2	2	1	3	4	2	0.1	0.2	1	0.1	0.1	1	3	10	4	1	2	2
	2.5	22	1	0.1	51	1	0.02	23	1	0.1	1	0.1	0.1	2	0.04	0.03	1	0.1	0.1	4	1	2
	5	21	0.8	0.04	26	0.6	0.03	46	2	0.04	4	0.2	0.1	4	0.1	0.02	2	0.1	0.02	4	2	0.7
mBA	0	9	8	1	3	5	2	8	12	2	0.6	0.8	2	4	1	0.5	4	20	9	10	10	3
	0.05	2	16	8	2	14	6	1	10	8	0.3	3	9	0.3	2	6	14	52	11	0.1	6	38
	0.5	6	6	1	5	3	0.9	6	6	1	1	1	1	0.6	0.5	0.9	19	30	3	1	3	2
	2.5	93	3	0.03	66	1	0.02	103	3	0.03	9	0.3	0.03	7	0.2	0.05	26	1	0.04	290	5	0.04
	5	80	2	0.02	67	1	0.02	70	2	0.03	15	0.4	0.03	10	0.2	0.03	8	0.2	0.03	154	6	0.09
AA	0	5	86	89	3	86	94	2	92	95	637	96	65	2	85	93	0.3	51	40	5	2	3
	0.05	5	57	25	4	43	24	2	26	21	3	65	22	2	51	23	9	41	21	0.1	4	10
	0.5	12	74	54	5	42	49	4	50	45	9	75	30	2	64	43	1004	74	20	6	6	3
	2.5	16	70	68	14	31	64	5	61	81	7	78	53	5	86	71	51	60	38	61	9	2
	5	6	60	73	5	36	71	4	41	80	4	72	58	1	66	68	56	39	36	55	7	1
CPrA	0	41	45	13	6	91	94	4	97	97	3	90	93	4	94	95	2	58	76	23	5	1
	0.05	72	28	3	13	86	81	9	93	90	3	68	84	1	69	78	0.6	25	27	3	5	3
	0.5	52	40	2	10	55	47	6	78	54	38	86	37	3	64	44	385	33	13	0.6	4	5
	2.5	111	25	2	17	25	49	9	71	68	40	63	23	4	69	53	323	45	28	57	6	2
	5	148	31	3	18	17	54	18	68	61	281	88	38	4	53	59	21	39	45	169	6	2
CBA	0	2	2	2	98	17	2	13	1	1	3	2	0.6	3	0.7	0.9	88	17	1	0.7	1	2
	0.05	7	20	12	2	8	8	4	0.4	0.2	0.2	3	7	0.5	0.4	0.4	590	81	3	0.2	0.7	4
	0.5	15	6	0.5	14	4	0.7	27	0.7	0.2	0.3	0.6	1	6	1	0.2	6386	81	2	0.3	1	5
	2.5	193	3	0.3	201	2	0.1	35	1	0.8	3	0.1	0.04	13	1.4	0.7	2219	55	1	17	2	0.3
	5	445	4	0.02	98	1	0.1	39	0.9	0.8	23	0.5	0.03	5	2	0.7	336	42	0.9	3	0.8	0.3
CPA	0	12	0.7	0.2	15	2	0.2	22	0.7	0.5	0.8	1	1	0.8	0.6	0.7	29	0.9	0.5	2	0.3	0.5
	0.05	6	0.8	0.1	4	0.6	0.1	19	0.5	0.04	0.8	0.2	0.1	2	0.3	0.2	51	1	0.1	2	0.9	0.4
	0.5	27	0.8	0.02	69	1	0.02	64	0.2	0.1	3	0.1	0.03	3	0.4	0.2	205	2	0.3	8	0.7	0.1
	2.5	20	0.4	0.03	37	0.5	0.02	23	0.3	0.1	2	0.1	0.1	3	0.4	0.2	151	2	0.3	32	2	0.1
	5	37	0.5	0.02	9	0.3	0.03	19	0.5	0.1	2	0.04	0.03	3	0.4	0.1	182	3	0.2	8	0.6	0.1
CHA	0	25	0.2	0.02	77	6	0.4	22	6	2	2	0.3	0.1	141	21	0.5	62	0.2	0.1	0.9	0.2	0.1
	0.05	15	0.2	0.02	108	2	0.04	64	3	0.2	0.6	0.03	0.02	1	0.5	0.3	53	1	0.1	0.4	0.1	0.2
	0.5	14	0.1	0.01	15	0.4	0.1	13	0.8	0.4	2	0.03	0.01	1	0.6	0.7	122	0.7	0.3	1	0.2	0.2
	2.5	7	0.1	0.01	6	0.3	0.1	6	0.7	0.5	2	0.04	0.01	2	1	0.6	37	0.9	0.3	2	0.2	0.2
	5	4	0.1	0.02	24	0.3	0.1	3	0.5	0.4	2	0.03	0.01	0.8	0.5	0.7	31	0.7	0.1	2	0.2	0.31

Conversion ratios (CR) were calculated to estimate the yield of each synthesis, both accelerated and bulk. The ratio represents the amount of material converted to form product and includes intermediates in the equation. CR were calculated using Equation S2 for each reaction. Each condition was analyzed in triplicate and the average CR are summarized in Table 1. Note: these values are estimated values of the yield and do not correct for differences in ionization efficiency.

## Results and discussion

**Figure 2:**
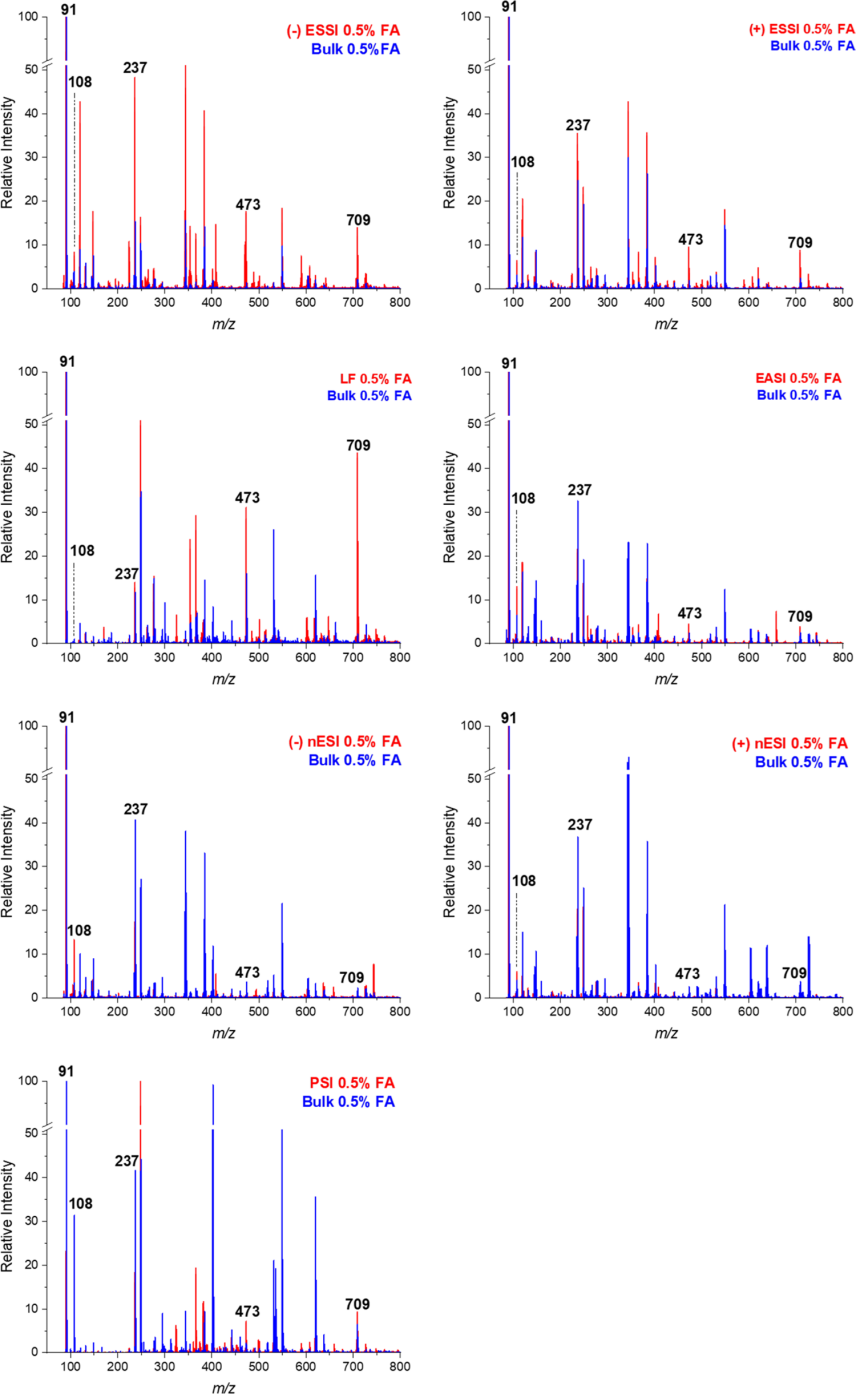
Comparison spectra for all five confined volume techniques including LF, PSI, −/+ ESSI, −/+ nESI, and EASI. Each technique shows a comparison between the accelerated technique (red) and the bulk reaction (blue). The reaction conditions were 10 mM glyoxal with 1% FA reacted with 20 mM BA (final concentration 5 mM glyoxal, 0.5% FA, and 10 mM BA). All spectra are relative to the base peak.

Traditionally, the condensation of glyoxal and BA forms HBIW, which creates the base cage structure of CL-20. The original reaction was used as a baseline to compare with the substituted amine reactions. In Fig. 2, all five acceleration techniques are compared using the BA reaction and one FA condition (0.5% FA). The spectrum for the accelerated technique (red) and the bulk reaction (blue) are overlaid to show a direct comparison of product formation. The apparent acceleration factors (AAF) are summarized in Table 1. Acceleration factors were calculated to compare the intensity ratios of product to starting material in both the accelerated technique and bulk (AAF = ((P)/(R))_droplet_/((P)/(R))_bulk_, where (P) is product intensity and (R) is reactant intensity). The starting material, BA, can be observed at *m/z* 108. However, the dominant peak is *m/z* 91 which is a fragment of BA after the loss of NH_3_ (corresponding to benzylium ion). Both peak intensities were considered when calculating acceleration factors. Two known intermediates involved in forming the cage structure were observed at *m/z* 237 and *m*/z 473 corresponding to a diimine structure with 1:2 glyoxal to BA and another diimine structure with 2:4 glyoxal to BA. HBIW was successfully formed in all five accelerated techniques and observed at *m/z* 709.

For Leidenfrost, the product peak at *m/z* 709 is ~ 43% relative to the starting material, whereas, in the bulk only ~ 2% of product is observed relative to the starting material. Since Leidenfrost is a heated confined volume system, to make a fair comparison, the bulk reaction was setup using a heated refluxing system. PSI is usually performed using Whatman 1 filter paper as the substrate, however, for this reaction, the starting material and product were not detected. This was possibly due to the reagents wicking into the paper and not being extracted from the pores. As an alternative, another substrate, Teslin^®^, was used for the PSI experiments, which allowed HBIW to be detected after the desired reaction time. Cellulose based paper substrates, such as Whatman 1 filter paper, are hydrophilic and have larger pore sizes compared to the Teslin^®^ substrate which is microporous and hydrophobic^54,55^. The reaction mixture was retained on the surface of the Teslin^®^ substrate due to its hydrophobicity allowing the reaction to occur in the thin film formed on the surface rather than diffusing into the paper when using the Whatman 1 filter paper and reacting in the pores. The combination of the thin film formed on the surface, and the reaction mixture being more readily wicked off the surface of the substrate led to the product being observed after switching to Teslin^®^.

For the spray-based techniques, when comparing EASI and ESSI, comparable product intensities are observed, implying that adding voltage to the spray system does not influence the formation of product. The polarity of the voltage, whether + 4 kV or − 4 kV was applied did not cause an effect on the amount of product formed. However, in nESI, very little product is observed compared to the other spray-based techniques. One difference in these methods is that desolvation gas is used in both ESSI and EASI, but not in nESI. The diameter of the spray emitter is also smaller in nESI, allowing for smaller droplets to be formed. The general trend observed in microdroplets is that acceleration increases as droplet size decreases, however, with such a large and complex cage structure, this reaction does not seem to follow this trend. With a larger droplet, there is a higher probability of having the correct mole ratio (3 glyoxal:6 BA) required for the reaction at the surface of the droplet and form the complex cage structure.

**Figure 3:**
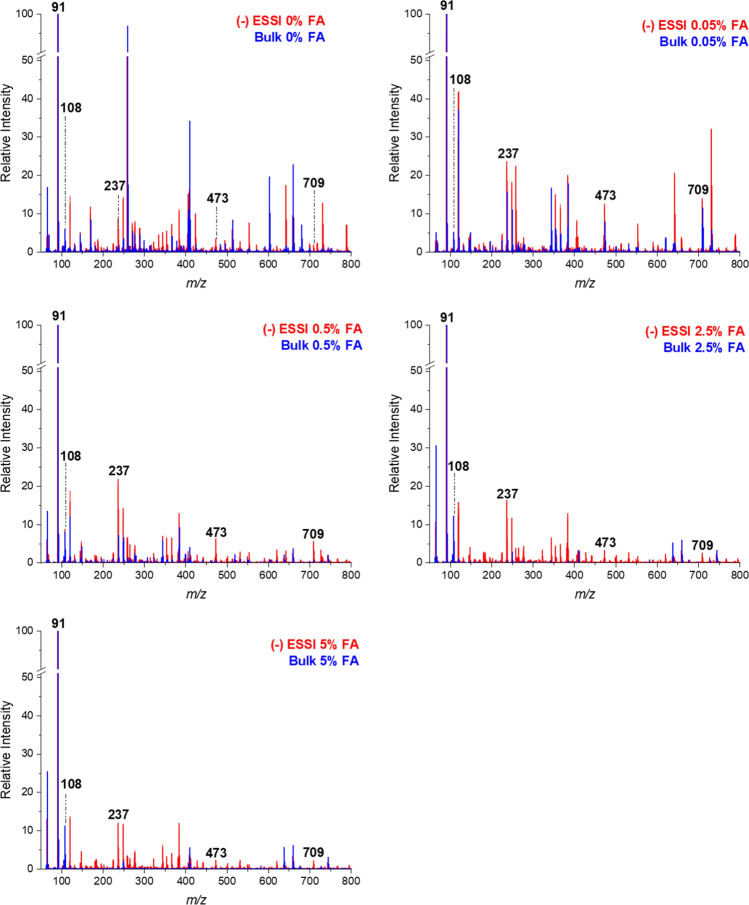
Overlaid mass spectra for the formation of HBIW (*m/z *709) using (−)ESSI. Spray (red) and bulk (blue) spectra are shown for all five FA conditions (0%, 0.05%, 0.5%, 2.5%, and 5%). Benzylium ion (*m/z* 91) is related to the starting material BA. Two intermediates were observed at *m/z* 237 and *m/z* 473. All spectra are relative to the base peak.

One advantage of performing accelerated reactions in confined volumes is the unique environment that microdroplets provide. Extreme pH values are commonly observed at the surface of these droplets helping to accelerate acid or base catalyzed reactions^56^. FA is used as the acid catalyst in this condensation reaction. Figure 3 shows the effect of varying the concentration of FA has on product formation in both spray and bulk. The spray technique, (−) ESSI, and other reactants (10 mM Glyoxal and 20 mM BA) are held constant for comparison. At 0% FA, the product at *m/z* 709 is observed in the spray method spectrum (red), however, it is not as abundant in the bulk spectrum (blue). The unique extreme pH environment of the droplet provides enough protons to catalyze the reaction without using FA as the catalyst. Forming the product in spray with no FA helps to eliminate one reagent which ultimately makes the reaction cheaper as well as reduces liquid waste. Although FA is not environmentally hazardous, this can translate to other reactions where the catalysts are toxic and reaction acceleration can be used as a greener option to eliminate the toxic reagent. The overlay spectra for the formic series comparison across the other accelerated techniques for BA and glyoxal are shown in Figs. S8–S13.

**Figure 4:**
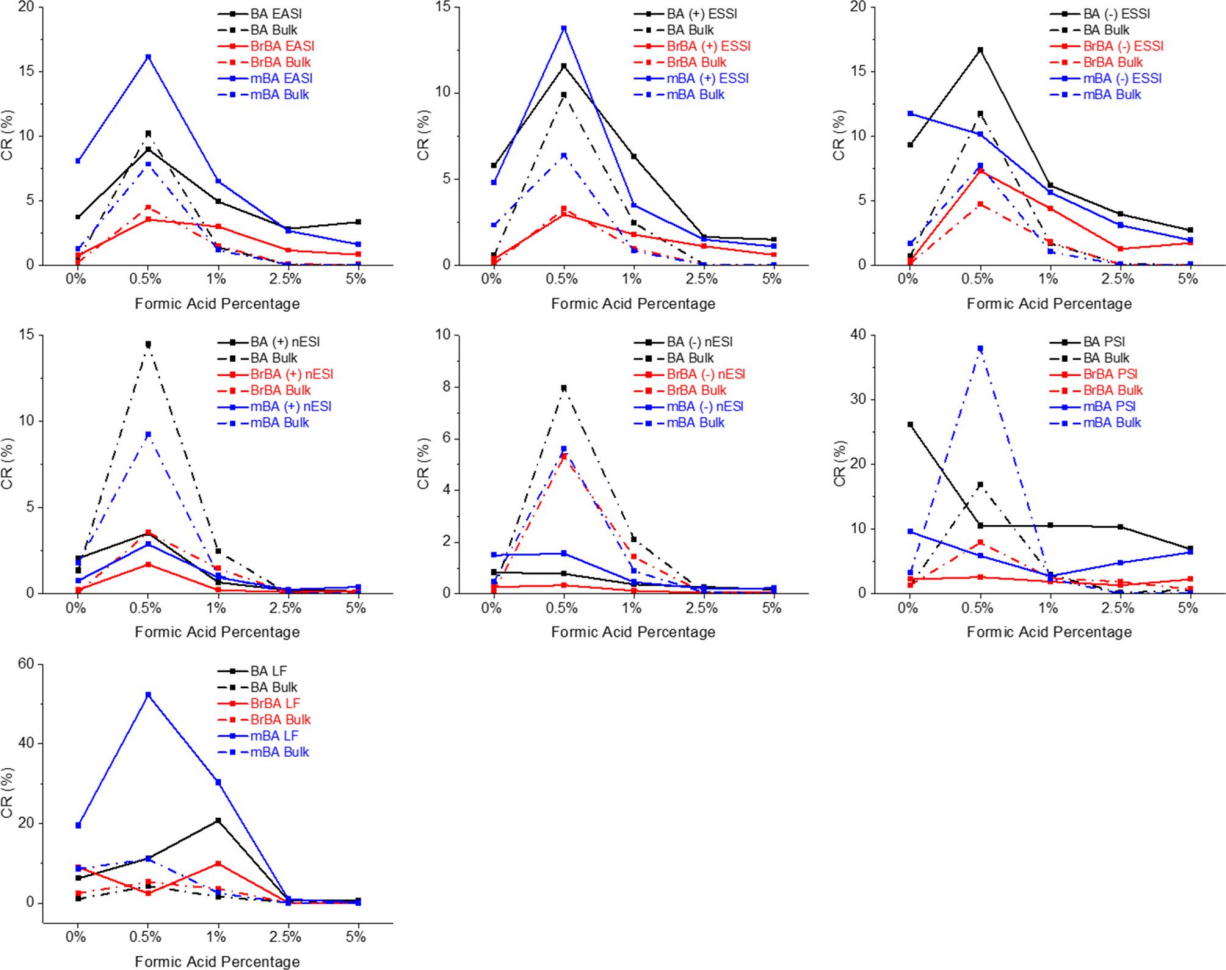
Average CRs for the substituted BA series, including BA (black), BrBA (red), and mBA (blue) are shown for all accelerated techniques. CR for both the accelerated techniques (solid line) and bulk techniques (dotted line) are compared.

In the overlay spectrum for 0.05% FA in Fig. 3, the bulk has a comparable product intensity to the (−) ESSI method. Across the substituted BA series, including BA, BrBA, and mBA, the highest product intensity for the bulk reactions occurs at 0.05% FA. Increasing the percentage of acid decreases the amount of product formed and hinders the reaction. This is seen more clearly in Fig. 4 where the conversion ratios (CR) are plotted against increasing percent FA. CR were calculated to provide a rough estimate for yield where CR = (P)/((R) + (I) + (P)). CR values account for intermediate (I) intensities as well^30,37^. The dotted lines represent the CR for each of the three amines in the substituted BA series. The highest CRs for bulk reactions are observed when the reaction mixture contains 0.05% FA. As the percent FA increases, the CR decreases meaning less reactants are converting to product. This is due to the excess of acid hindering the product formation. When comparing the CR of confined volume systems, 0.05% FA overall has the highest CR with a few exceptions, primarily at 0% FA which again is due to the unique pH environment of the microdroplets. The CR trends of the rapid confined volume methodologies are correlative to the solution phase bulks and can be applied to guide optimal reaction conditions for the bulk solution phase reactions in a rapid manner.

**Figure 5:**
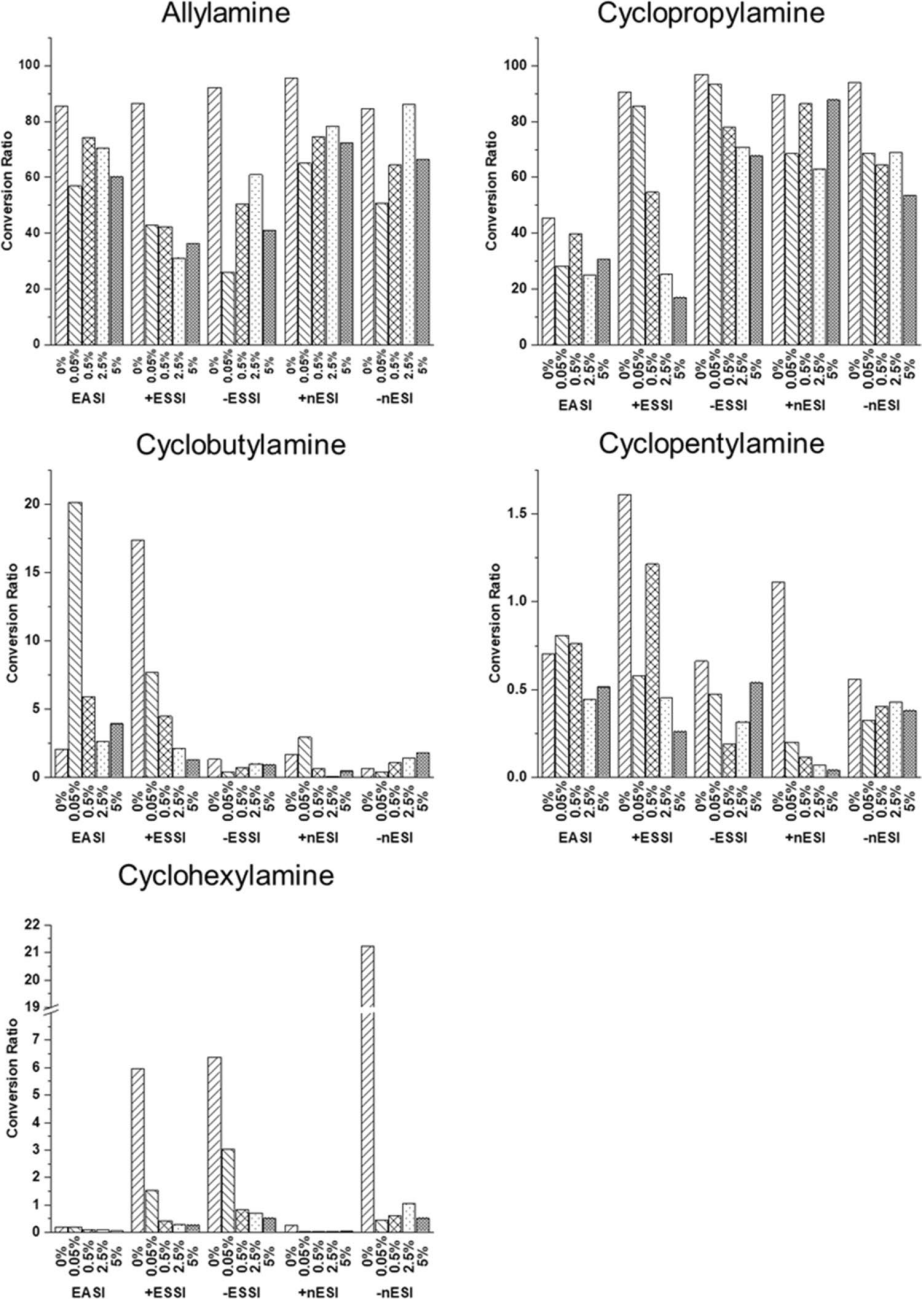
Comparison of CRs for additional amine analogues across all the spray-based techniques.

Accelerated confined volume methodologies can also be applied to screen alternative starting materials, such as the substituted amines, to determine if the reaction will form the desired product before running the equivalent bulk solution phase reaction. The accelerated techniques screened AA, CPrA, CBA, CPA, and CHA to examine their potential as replacements for BA. Figure 5 compares the CR for the spray-based techniques across all amines. Overall, the CR are better for AA and CPrA, of which AA has been previously reported in the literature to form the cage structure^57,58^. The larger amines in the series (CBA, CPA, and CHA) show a drastic drop in CR (< 20% conversion). As a screening method for finding a substitute for BA, reaction acceleration can help determine not only if the product forms, but which amines are better at forming the product. This ultimately narrows down the possible replacement candidates before scaling up solution phase bulk reactions, while enabling a higher-throughput screening methodology. The higher CRs in this series are also higher for the lower percentages of FA and tend to drop off as the percentage increases. This also follows the general trend of helping to reduce the amount of reagents used with the lower FA acid needed for the reaction. At the lower percentages, more reactant is converted to product, so these conditions would also be ideal for the multiplexed sprayer setup to produce the maximum amount of product with spray-based methods. Conversion ratios for LF and PSI for the substituted amines are shown in Fig. S14.

## Conclusions

Reaction acceleration techniques were used to explore the condensation reaction of glyoxal and BA to form HBIW, a precursor to CL-20 with a complex cage structure. Acceleration of the product was observed in microdroplets, thin films, and Leidenfrost droplets. For microdroplet techniques, adding high voltage to the system did not influence the product formation, however, the nebulizing gas, as well as the overall droplet size did affect product formation, with the smaller droplets and lack of nebulizing gas in nESI consistently formed less product compared to the larger droplets with nebulizing gas in ESSI and EASI. Using accelerated confined volume techniques also allows for the complete removal of acid catalyst from the reaction. The unique droplet environment creates a pH gradient low enough to form product without FA. Eliminating one reagent (i.e. the acid catalyst) can help to reduce liquid waste and make the overall reaction cheaper, if conducting larger scale or multiplexed confined volume systems for synthesis. Larger reaction acceleration apparatus and methods need to be explored to determine if confined volume techniques can be utilized for practical applications. CRs were calculated for each reaction to determine approximate yields and the values obtained by the confined volume methods can be utilized to estimate which conditions would be ideal for bulk solution phase reactions. This was demonstrated with BA, where the ideal conditions in bulk and the confined volume systems were both 0.05% FA. Finally, the CR values from confined volume systems can be used to screen novel reactants to improve synthetic schemes.

## Supplementary Information

Supplementary Information.
