# Supplementary material for: Accelerated synthesis of energetic precursor cage compounds using confined volume systems
Source: Sci Rep. 2021 Dec 16;11:24093. doi: 10.1038/s41598-021-02945-1 (PMC8677777; doi:10.1038/s41598-021-02945-1)
Supplement: Supplementary file 1 — Supplementary Information. [file 41598_2021_2945_MOESM1_ESM.pdf]

Supporting Information

Accelerated Synthesis of Energetic Precursor Cage Compounds using Confined Volume Systems

Hilary M. Brown,[a] Karan R. Doppalapudi,[a] and Patrick W. Fedick*[a]

[a] Chemistry Division, Naval Air Warfare Center Weapons Division (NAWCWD), United States Navy Naval Air Systems Command (NAVAIR), China Lake, California 93555, USA.

* Corresponding author

Table of Contents

Experimental 3

Calculation of Apparent Acceleration Factor 3

Calculation of Conversion Ratio 3

Accelerated Techniques 4

Easy ambient sonic-spray ionization (EASI) and Electrosonic spray ionization (ESSI) 4

Nano-electrospray ionization (nESI) 5

Paper Spray Ionization (PSI) 6

Leidenfrost (LF) 7

Supplemental Figures 8

Figure S8. EASI versus bulk formic series for HBIW reaction 8

Figure S9. (+) ESSI versus bulk formic series for HBIW reaction 9

Figure S10. (-) nESI versus bulk formic series for HBIW reaction 10

Figure S11. (+) nESI versus bulk formic series for HBIW reaction 11

Figure S12. PSI versus bulk formic series for HBIW reaction 12

Figure S13. LF versus bulk formic series for HBIW reaction 13

Figure S14. Conversion ratios for amine series including LF and PSI 14

References 15

Experimental

Calculation of Apparent Acceleration Factor

$$AAF = \frac{\left(\frac{\text{Product Intensity}}{\text{Reactant Intensity}}\right)_{droplet}}{\left(\frac{\text{Product Intensity}}{\text{Reactant Intensity}}\right)_{bulk}} = \frac{\left(\frac{\text{P}}{\text{R}}\right)_{droplet}}{\left(\frac{\text{P}}{\text{R}}\right)_{bulk}}$$

Equation S1. Equation used to calculate the apparent acceleration factor (AAF) for each reaction setup. Compares the intensity of the product and reactants in spray to the intensity of products and reactants in bulk.

Calculation of Conversion Ratio

$$CR = \frac{\text{Product Intensity}}{(Reactant\ Intensity + Intermediate\ Intensity + Product\ Intensity)} = \frac{P}{(R + I + P)}$$

Equation S2. Equation used to calculate conversion ratios (CR).

Accelerated Techniques

Easy ambient sonic-spray ionization (EASI) and Electrosonic spray ionization (ESSI)

A custom ESI source was built using fused silica capillary (IDEX, 100µm IDx360µmOD), green capillary sleeves (IDEX, 1/16"ODx0.0155"ID and 1/16"ODx0.033"ID), union assembly (PEEK, 10-32 Coned), brass tee union (Swagelok, 1/16"), and stainless-steel tubing (IDEX, 1/16"ODx0.020IDx5cm, yellow band). **Figure S1** below shows the electrospray source assembly including part descriptions. A PHD Ultra syringe pump (Harvard Apparatus) and a 500 µL 1750 gas tight syringe (Hamilton) were used to push the reaction mixture through the capillary at 50 µL/min for a 10-minute reaction. For both EASI and ESSI, the N_2 gas pressure was 100 psi. ESSI voltage was applied using an external high voltage power supply (Bertan, Model 205B-10R) set to 4kV for both positive and negative experiments. Product was collected on glass wool inside a 15 mL conical tube (spray distance 7.5cm). **Figure S2** and **S3** shows the setup used for both EASI and ESSI, respectively. Product was extracted from the using 10 mL acetonitrile (ACN). Bulk reaction mixtures (500 µL) reacted in glass vials for 10 minutes and were then added to 10 mL of ACN.

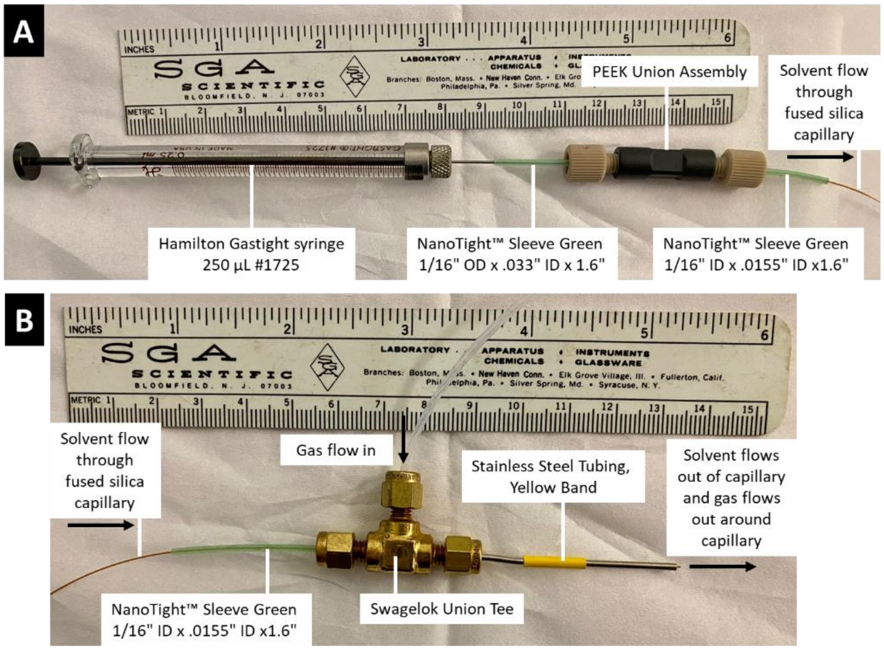

Figure S1. (A) Syringe assembly for spray-based techniques. (B) Electrospray source assembly.

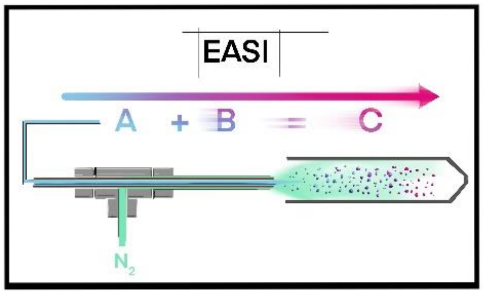

Figure S2. EASI setup, sprayer is directed into a conical tube where reaction mixture is sprayed and collected on glass wool. Spray distance is 7.5 cm from the tip of the sprayer to the glass wool. EASI has gas flow only.

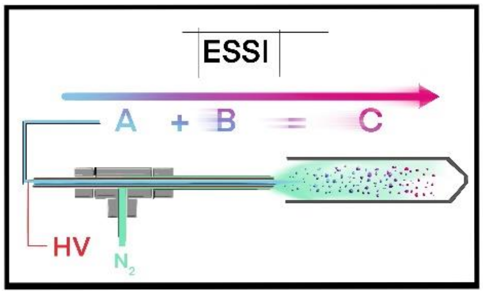

Figure S3. ESSI setup, sprayer is directed into a conical tube where reaction mixture is sprayed and collected on glass wool. Spray distance is 7.5 cm from the tip of the sprayer to the glass wool. ESSI has gas flow and high voltage applied.

Nano-electrospray ionization (nESI)

A custom nano-electrospray (nESI) source was constructed using fused silica capillary (IDEX, 100µmIDx360µmOD), a conductive microunion assembly (IDEX), copper wire, and a 100µm PicoTip™ nanospray emitter (TaperTip™, New Objective). A picture of the nESI source is shown in **Figure S4** including part labels. The reaction mixture was pumped through the capillary at 25 µL/min for a 10-minute reaction. The nESI voltage was set to 3.5kV for both positive and negative experiments. Product was collected on glass wool atop a stainless-steel L-bracket that was electrically grounded. **Figure S5** shows a visual representation of the nESI setup. Product was extracted from glass wool using 10 mL ACN. Bulk reaction mixtures (500 µL) reacted in glass vials for 10 minutes and were then added to 10 mL of ACN.

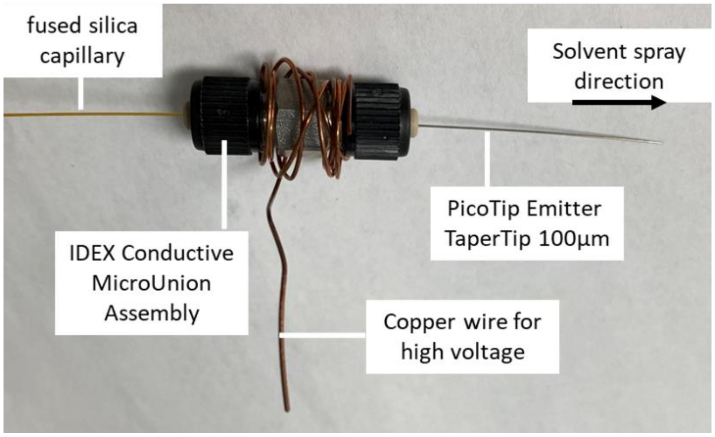

Figure S4. nESI source assembly. Copper wire was wrapped around the conductive union to apply high voltage.

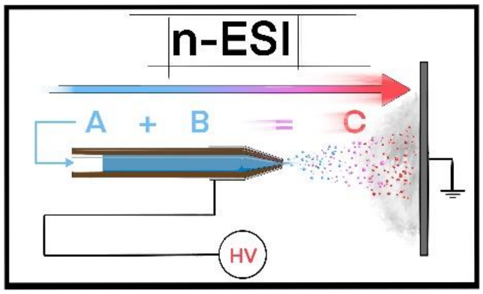

Figure S5. nESI setup, sprayer is directed at glass wool sitting on a stainless-steel L-bracket. The L-bracket was electrically grounded. Spray distance is 7.5 cm from the tip of the sprayer to the glass wool. nESI is has high voltage only.

Paper Spray Ionization (PSI)

Paper spray experiments were performed using PPG Teslin® SP600 substrate. The Teslin® substrate is held in position using a ring stand and a copper clip attached to the instrument's high voltage supply. 10 µL each of glyoxal and amine solutions were spotted onto the substrate and allowed to dry for 10 minutes. Once dry, the paper was positioned in front of the mass spectrometer inlet. ACN (30 µL) was added, and high voltage (4kV) was applied to create the spray plume. For bulk reactions, the reaction mixture was mixed in a vial and allowed to react for 10 minutes. 20 µL was spotted on the substrate but not allowed to dry. ACN and high voltage were immediately applied. Once the spray was formed, spectra were collected for each reaction condition. **Figure S6** shows a visual representation of the PSI setup.

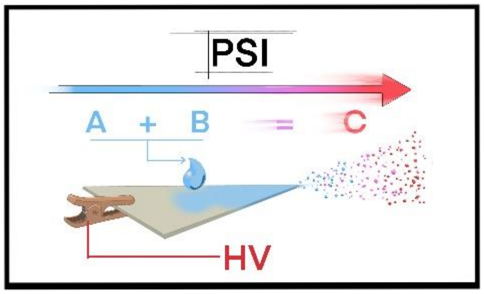

Figure S6. PSI setup. High voltage is applied using a copper clip that also holds the paper substrate in front of the mass spectrometer inlet. ACN was used as the spray solvent.

Leidenfrost (LF)

Leidenfrost experiments were setup similarly to those previously described with slight modifications.[1] Briefly, a glass spot plate (Pyrex, 7220-85) was set on a hot plate (Fisher, Isotemp) maintained at 540°C. **Figure S7** shows a visual representation of the droplet in the glass well used for LF experiments. The reaction mixture (500 µL) was added dropwise to the well to form the initial droplet. ACN was constantly supplied (2.5 mL syringe, 180 µL/min) to the well for the duration of the reaction to replenish evaporated solvent. The droplet was maintained for 10-minutes. Droplets collected from Leidenfrost experiments were collected and added to 10 mL of ACN. Bulk reaction mixtures (500 µL) were added to a round bottom flask and refluxed for 10 minutes at 200°C. The resulting solution was then added to 10 mL of ACN.

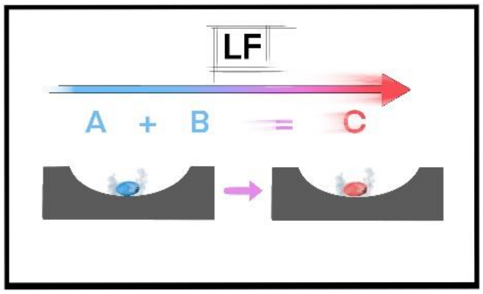

Figure S7. Leidenfrost setup. Well plate was heated to 540°C. Reaction droplet was maintained in the well for 10 minutes by adding ACN at a constant rate to replace evaporated solvent.

Supplemental Figures

Figure S8. EASI versus bulk formic series for HBIW reaction

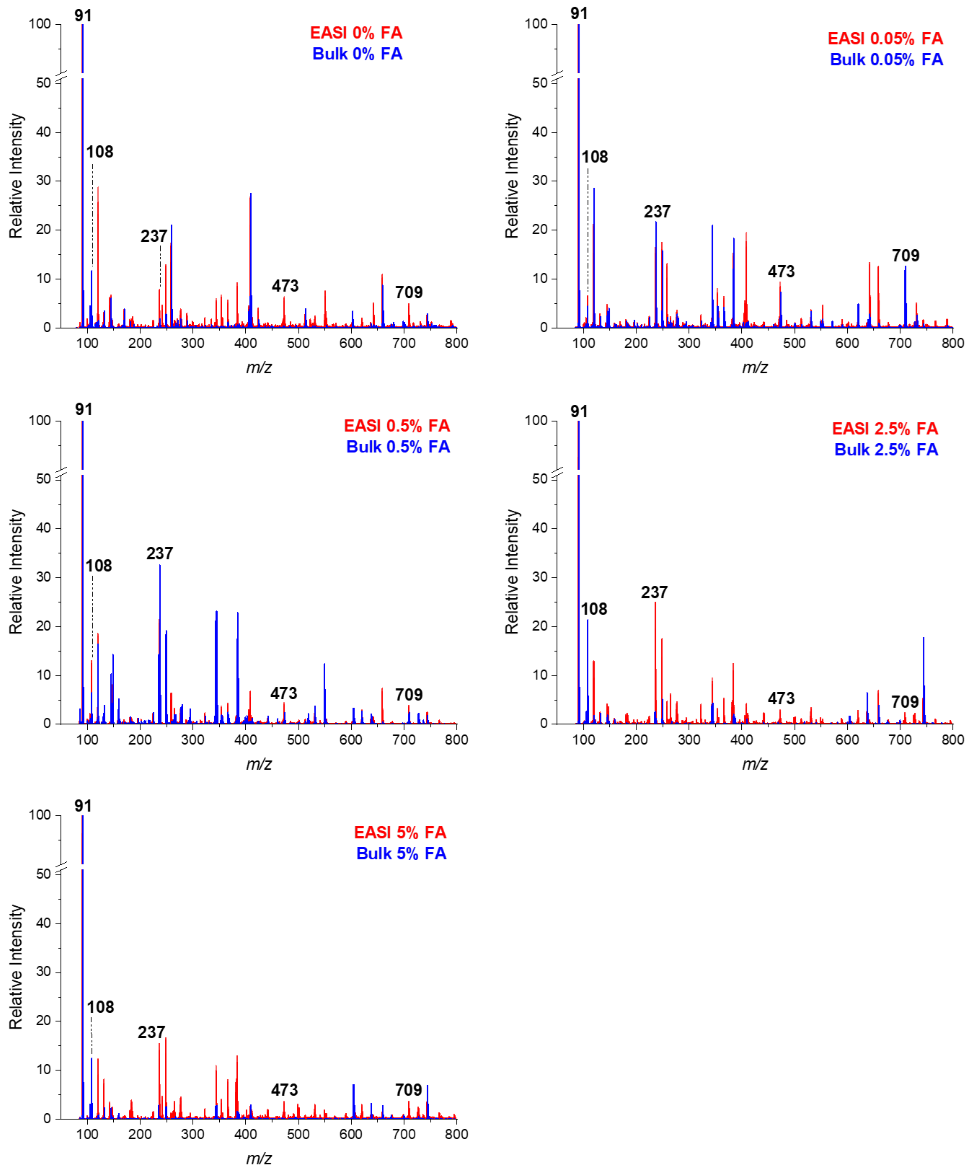

Figure S8. Formic acid series for Benzylamine and Glyoxal reaction using EASI. Bulk (blue) and EASI (red) spectra are overlaid for comparison. Spectra are relative to base peak.

Figure S9. (+) ESSI versus bulk formic series for HBIW reaction

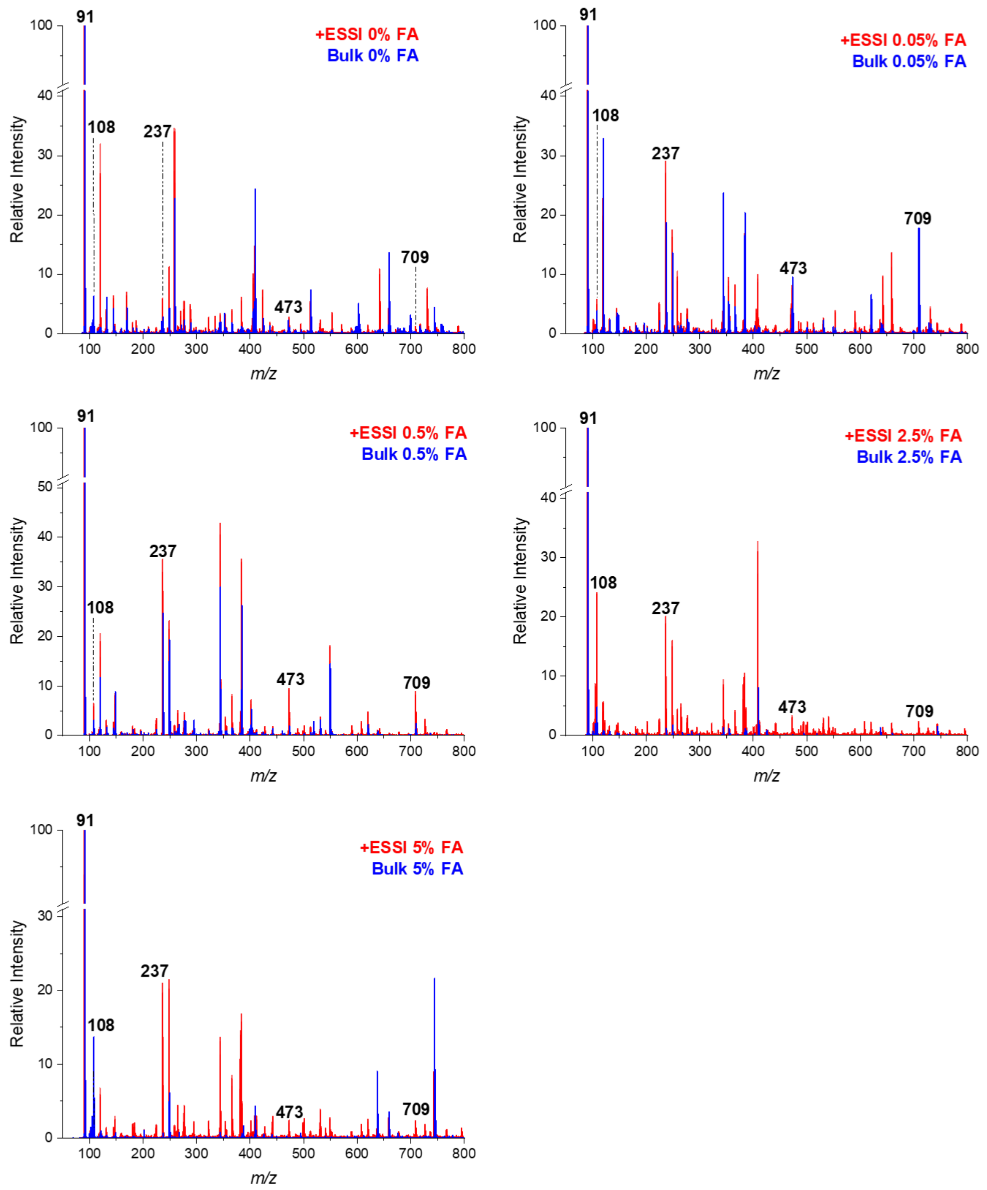

Figure S9. Formic acid series for Benzylamine and Glyoxal reaction using (+) ESSI. Bulk (blue) and (+) ESSI (red) spectra are overlaid for comparison. Spectra are relative to base peak.

Figure S10. (-) nESI versus bulk formic series for HBIW reaction

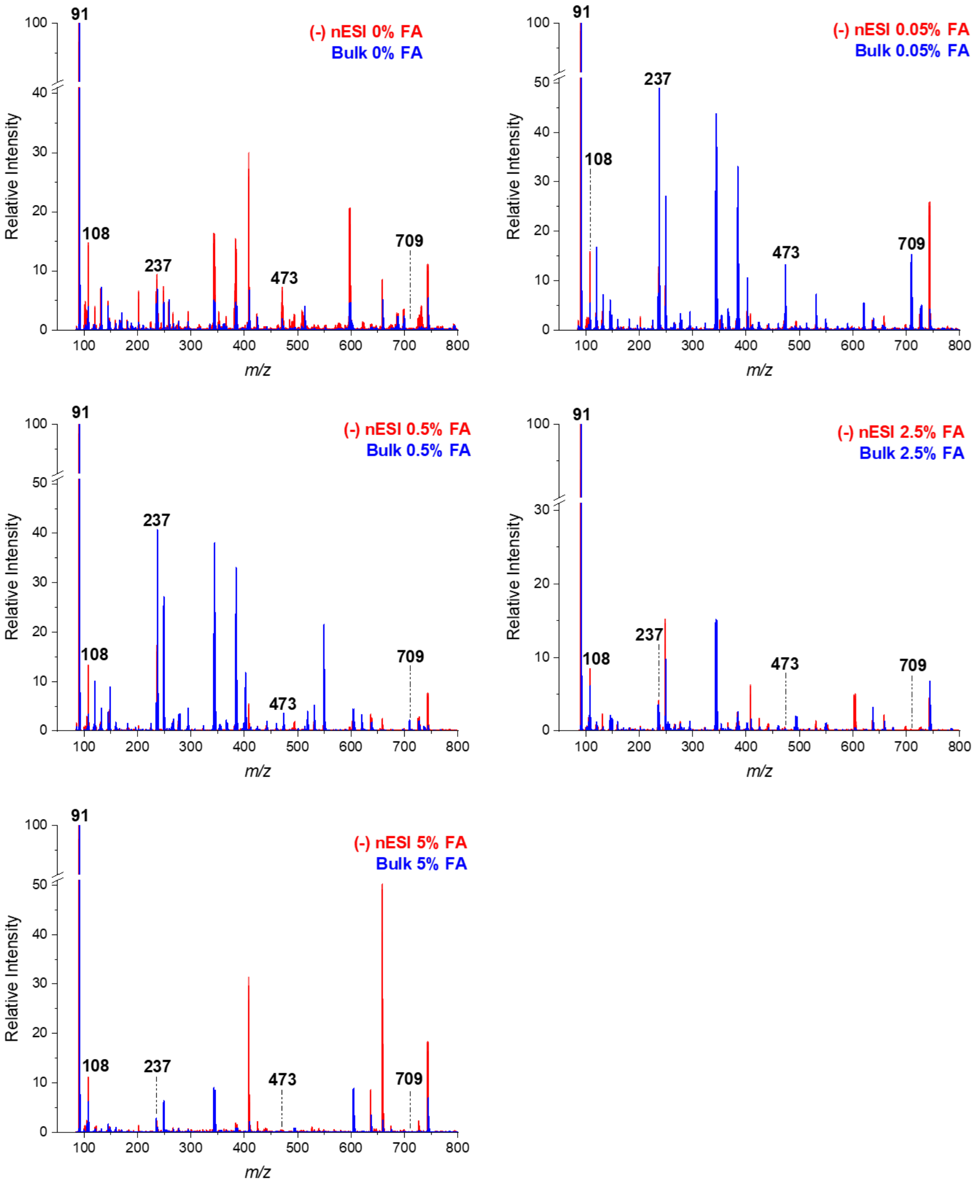

Figure S10. Formic acid series for Benzylamine and Glyoxal reaction using (-) nESI. Bulk (blue) and (-) nESI (red) spectra are overlaid for comparison. Spectra are relative to base peak.

Figure S11. (+) nESI versus bulk formic series for HBIW reaction

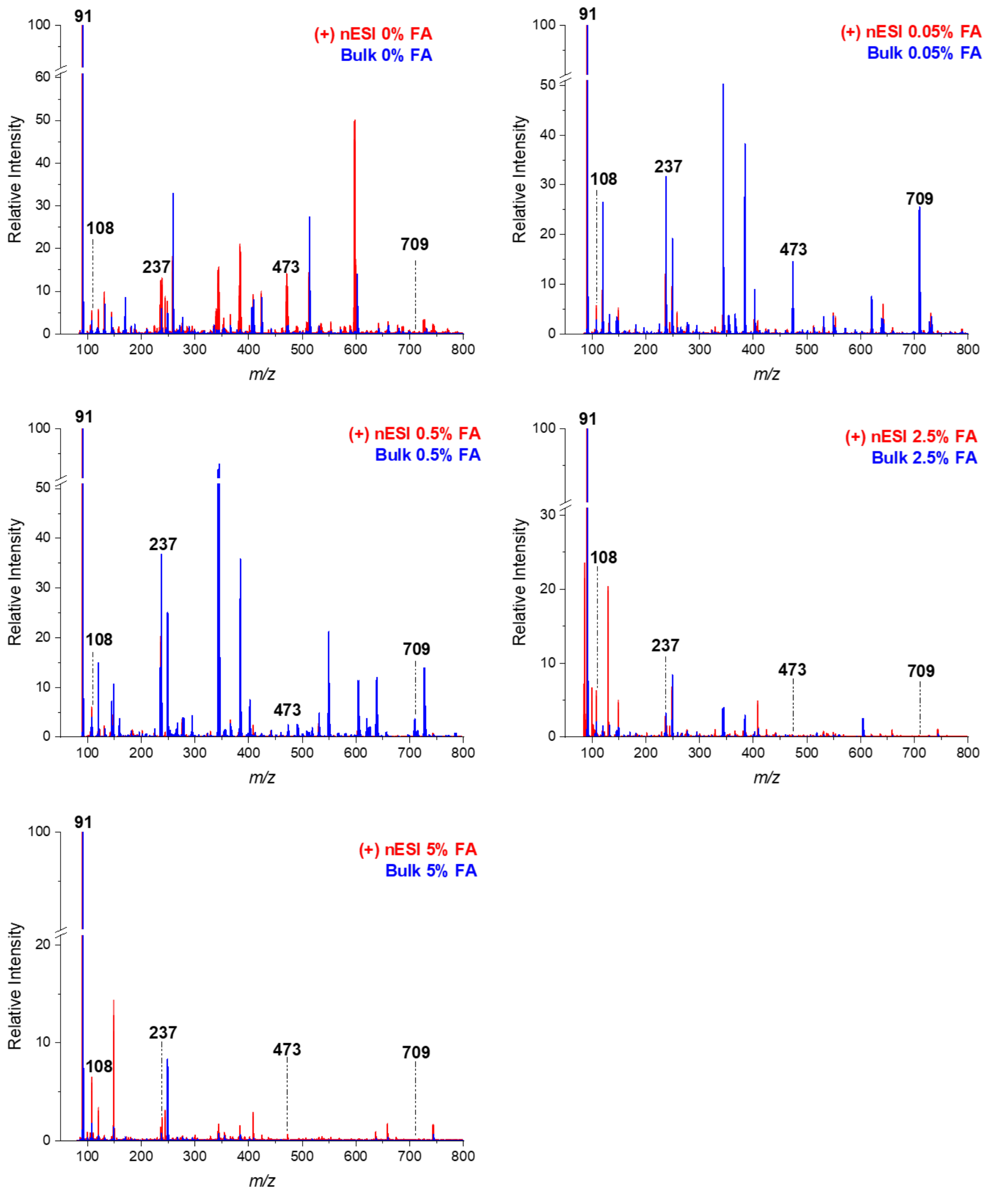

Figure S11. Formic acid series for Benzylamine and Glyoxal reaction using (+) nESI. Bulk (blue) and (+) nESI (red) spectra are overlaid for comparison. Spectra are relative to base peak.

Figure S12. PSI versus bulk formic series for HBIW reaction

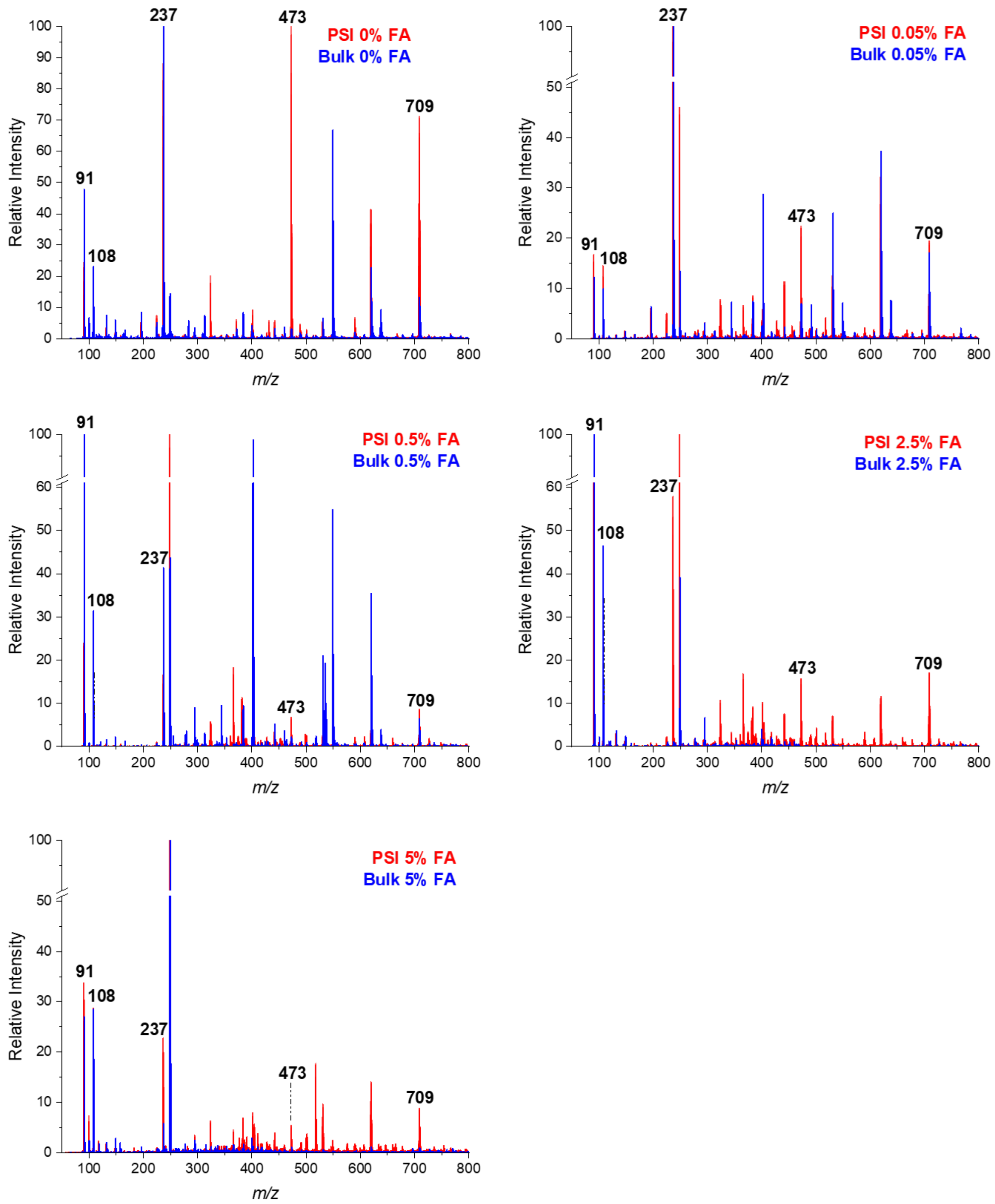

Figure S12. Formic acid series for Benzylamine and Glyoxal reaction using PSI. Bulk (blue) and PSI (red) spectra are overlaid for comparison. Spectra are relative to base peak.

Figure S13. LF versus bulk formic series for HBIW reaction

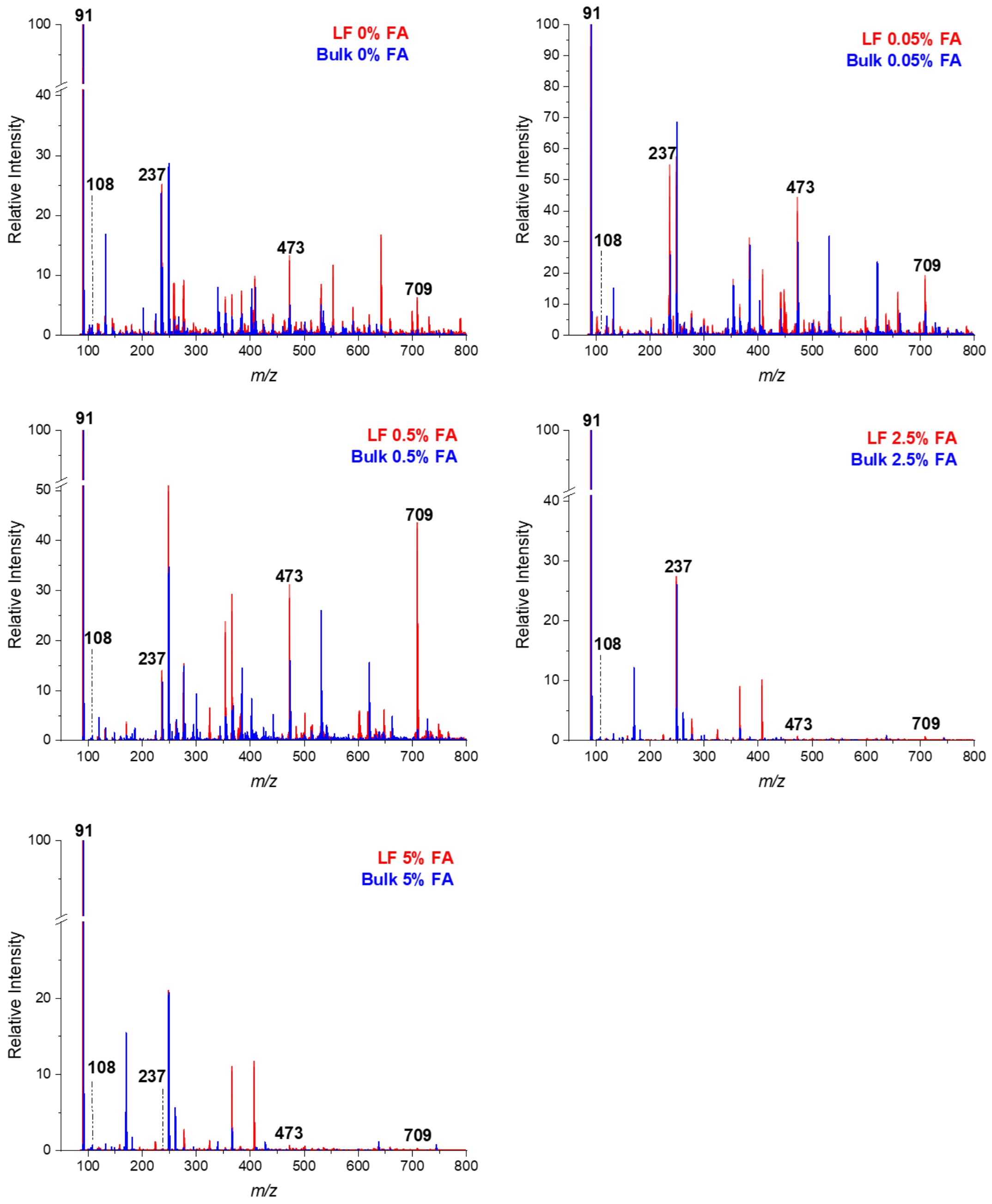

Figure S13. Formic acid series for Benzylamine and Glyoxal reaction using LF. Bulk (blue) and LF (red) spectra are overlaid for comparison. Spectra are relative to base peak.

Figure S14. Conversion ratios for amine series including LF and PSI

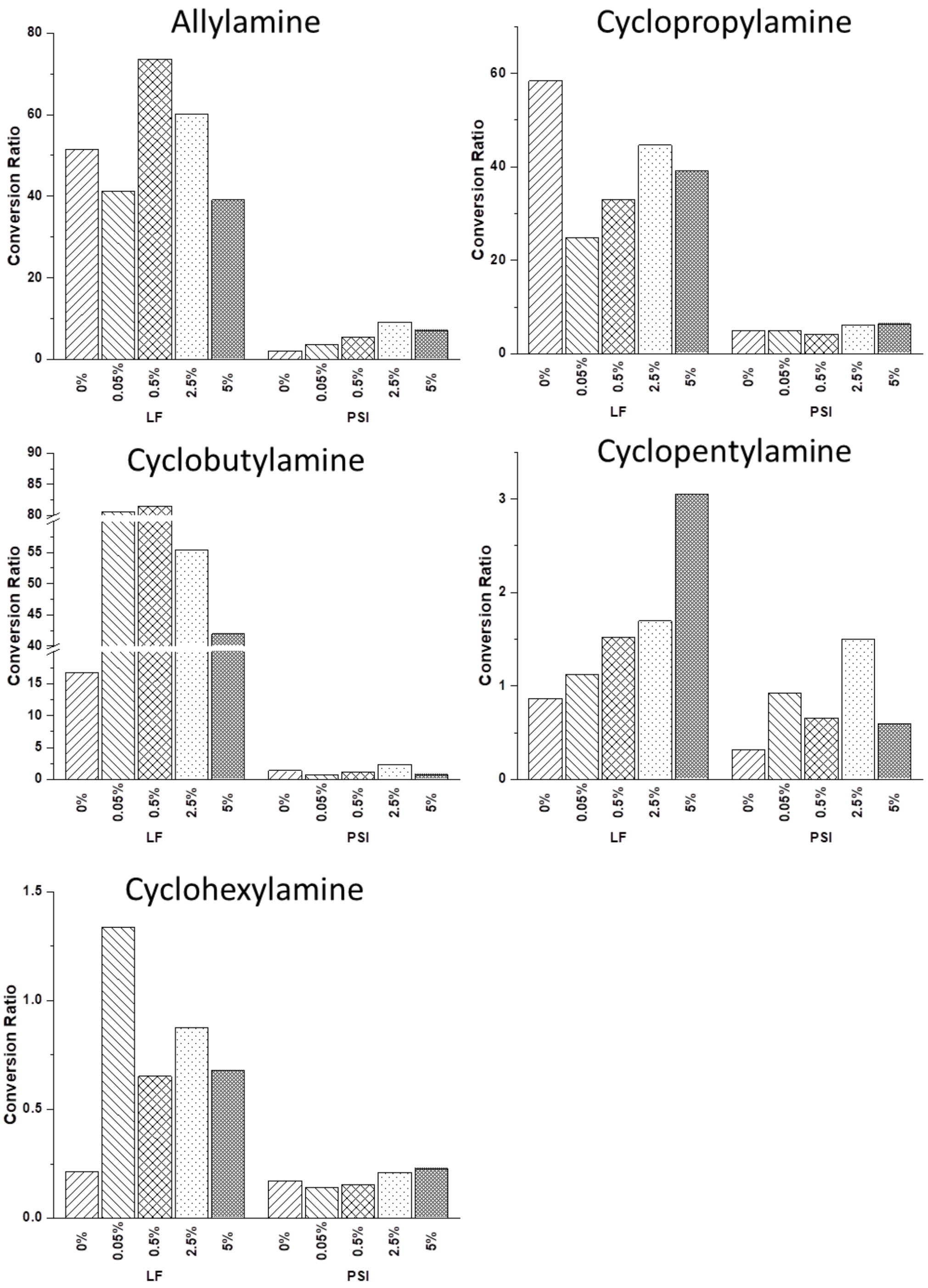

Figure S14. Comparison of CRs for additional amine analogues for LF and PSI.

References

[1] aY. Li, Y. Liu, H. Gao, R. Helmy, W. P. Wuelfing, C. J. Welch, R. G. Cooks, *Chemistry – A European Journal* **2018**, *24*, 7349-7353; bR. M. Bain, C. J. Pulliam, F. Thery, R. G. Cooks, *Angewandte Chemie International Edition* **2016**, *55*, 10478-10482; cP. W. Fedick, K. Iyer, Z. Wei, L. Avramova, G. O. Capek, R. G. Cooks, *Journal of The American Society for Mass Spectrometry* **2019**, *30*, 2144-2151.
